# Assessment of Hypokalemia and Clinical Characteristics in Patients With Coronavirus Disease 2019 in Wenzhou, China

**DOI:** 10.1001/jamanetworkopen.2020.11122

**Published:** 2020-06-11

**Authors:** Dong Chen, Xiaokun Li, Qifa Song, Chenchan Hu, Feifei Su, Jianyi Dai, Yinghai Ye, Jianping Huang, Xiaoming Zhang

**Affiliations:** 1 Department of Infectious Diseases, The Ding Li Clinical College of Wenzhou Medical University, Wenzhou, Zhejiang Province, China; 2 Department of Infectious Diseases, Sixth People’s Hospital of Wenzhou, Wenzhou, Zhejiang Province, China; 3 Virus Research Institute, Wenzhou Medical University, Wenzhou, Zhejiang Province, China; 4 Department of Microbiology, Ningbo Municipal Centre for Disease Control and Prevention, Ningbo, Zhejiang Province, China; 5 Department of Microbiology, Wenzhou Municipal Centre for Disease Control and Prevention, Wenzhou, Zhejiang Province, China

## Abstract

**Importance:**

Severe acute respiratory syndrome coronavirus 2 has caused a global outbreak of coronavirus disease 2019 (COVID-19). Severe acute respiratory syndrome coronavirus 2 binds angiotensin-converting enzyme 2 of the rennin-angiotensin system, resulting in hypokalemia.

**Objective:**

To investigate the prevalence, causes, and clinical implications of hypokalemia, including its possible association with treatment outcomes, among patients with COVID-19.

**Design, Setting, and Participants:**

This cohort study was conducted at Wenzhou Central Hospital and Sixth People’s Hospital of Wenzhou, Wenzhou, China, from January 11, 2020, to February 15, 2020. Participants included patients who received a diagnosis of COVID-19 according to the criteria issued by the Chinese Health Bureau and were admitted to the hospital. The patients were classified as having severe hypokalemia (plasma potassium <3 mmol/L), hypokalemia (plasma potassium 3-3.5 mmol/L), and normokalemia (plasma potassium >3.5 mmol/L). The clinical features, therapy, and outcomes were compared between the 3 groups. Data analysis was conducted in March 2020.

**Interventions:**

The patients were given general support and antiviral therapy. Their epidemiological and clinical features were collected.

**Main Outcomes and Measures:**

The prevalence of hypokalemia and response to treatment with potassium supplements were measured by analyzing plasma and urine potassium levels.

**Results:**

One hundred seventy-five patients (87 female patients [50%]; mean [SD] age, 45 [14] years) were classified as having severe hypokalemia (31 patients [18%]), hypokalemia (64 patients [37%]), and normokalemia (80 patients [46%]). Patients with severe hypokalemia had statistically significantly higher body temperature (mean [SD], 37.6 °C [0.9 °C]) than the patients with hypokalemia (mean [SD], 37.2 °C [0.7 °C]; difference, 0.4 °C; 95% CI, 0.2-0.6 °C; *P* = .02) and the patients with normokalemia (mean [SD], 37.1 °C [0.8 °C]; difference, 0.5 °C; 95% CI, 0.3-0.7 °C; *P* = .005). Patients with higher levels of hypokalemia also had higher creatine kinase levels (severe hypokalemia, mean [SD], 200 [257] U/L [median, 113 U/L; interquartile range {IQR}, 61-242 U/L]; hypokalemia, mean [SD], 97 [85] U/L; and normokalemia, mean [SD], 82 [57] U/L), higher creatine kinase–MB fraction (severe hypokalemia, mean [SD], 32 [39] U/L [median, 14 U/L; IQR, 11-36 U/L]; hypokalemia, mean [SD], 18 [15] U/L; and normokalemia, mean [SD], 15 [8] U/L), higher lactate dehydrogenase levels (mean [SD], severe hypokalemia, 256 [88] U/L; hypokalemia, 212 [59] U/L; and normokalemia, 199 [61] U/L), and higher C-reactive protein levels (severe hypokalemia, mean [SD], 29 [23] mg/L; hypokalemia, mean [SD], 18 [20] mg/L [median, 12, mg/L; IQR, 4-25 mg/L]; and normokalemia, mean [SD], 15 [18] mg/L [median, 6 U/L; IQR, 3-17 U/L]). Of 40 severely and critically ill patients, 34 (85%) had hypokalemia. Patients with severe hypokalemia were given potassium at a dose of 40 mEq per day, for a total mean (SD) of 453 (53) mEq potassium chloride, during the hospital stay. The patients responded well to potassium supplements as they recovered.

**Conclusions and Relevance:**

The correction of hypokalemia is challenging because of continuous renal potassium loss resulting from the degradation of angiotensin-converting enzyme 2. The high prevalence of hypokalemia among patients with COVID-19 suggests the presence of disordered rennin-angiotensin system activity, which increases as a result of reduced counteractivity of angiotensin-converting enzyme 2, which is bound by severe acute respiratory syndrome coronavirus 2.

## Key Points

**Question:**

What is the prevalence of hypokalemia among patients with coronavirus disease 2019 and is it associated with treatment outcomes?

**Findings:**

In this cohort study, hypokalemia was prevalent among patients with coronavirus disease 2019 and was associated with disease severity. It was challenging to correct hypokalemia because of the continuous renal potassium loss.

**Meaning:**

The high prevalence of hypokalemia among patients with coronavirus disease 2019 suggests the presence of disordered rennin-angiotensin system activity, which is increased as a result of the reduced counteractivity of angiotensin-converting enzyme 2, which is bound by severe acute respiratory syndrome coronavirus 2.

## Teaser

This cohort study investigates the prevalence, causes, and clinical implications of hypokalemia, including its possible association with treatment outcomes, among patients with coronavirus disease 2019 (COVID-19) in Wenzhou, China.

## Introduction

Since December 2019, severe acute respiratory syndrome coronavirus 2 (SARS-CoV-2) has caused an outbreak of coronavirus disease 2019 (COVID-19) in the city Wuhan of China.^1^ The virus invades human cells by binding angiotensin-converting enzyme 2 (ACE2) on a cell membrane^2^ that is widely distributed in many human vital organs, such as heart, liver, kidney, and lungs.^3^ Angiotensin-converting enzyme 2 is the principal counter-regulatory mechanism for the main axis of the rennin-angiotensin system (RAS), which is critical in the control of blood pressure and electrolyte balance.^4^ SARS-CoV-2 binds ACE2 and enhances the degradation of ACE2 and, thus, decreases the counteraction of ACE2 on RAS. This leads to increased reabsorption of sodium and water, thereby increasing blood pressure and excretion of potassium (K^+^).^5^ In addition, patients with COVID-19 often have gastrointestinal symptoms, such as diarrhea and vomiting.^6^ Collectively, the effects of COVID-19 on RAS and the gastrointestinal system probably lead to disruptions of homeostasis of electrolytes and pH.

One of the disruptions that may reflect the progression of COVID-19 and should be closely monitored is hypokalemia. Hypokalemia results in cellular hyperpolarity, increases resting potential, and hastens depolarization in cardiac cells and lung cells.^7^ Severe hypokalemia (ie, <3 mmol/L plasma K^+^) can trigger ventricular arrhythmia and respiratory muscle dysfunction, both of which are life-threatening conditions in patients with severe COVID-19. This implies that hypokalemia may be associated with treatment outcomes for patients with COVID-19 and should be seriously addressed because these patients have a high prevalence of dysfunction in the heart, lungs, and other vital organs.

Because little is known about the prevalence of hypokalemia and its association with treatment outcomes for patients with COVID-19, here we report the high prevalence of hypokalemia in such patients and investigate the possible causes and clinical meaning. We also examine the possible association of hypokalemia with treatment outcomes for these patients.

## Methods

### Study Population

This study was reviewed and approved by the ethics committees of Wenzhou Central Hospital and Sixth People’s Hospital of Wenzhou. Oral informed consent was obtained from all patients. This study follows the Strengthening the Reporting of Observational Studies in Epidemiology (STROBE) reporting guideline.

We included patients with COVID-19 who were admitted to the hospital in Wenzhou, China, from January 11, 2020, to February 15, 2020. All patients were aged older than 14 years and received a diagnosis of COVID-19 according to the criteria issued by the National Health and Health Commission of the People’s Republic of China.^8^ Their clinical characteristics have not been previously reported. All cases were screened according to the presence of cough, fever, and radiographic presentations and were confirmed by reverse transcriptase–polymerase chain reaction on respiratory tract samples to test for a sequence of SARS-CoV-2. We categorized the patients as having 4 levels of disease severity, as follows: mild cases involved mild clinical manifestations and no pneumonia; moderate cases involved respiratory symptoms and mild pneumonia; severe cases involved respiratory distress (≥30 breaths/min), oxygen saturation (≤93% at rest), or arterial partial pressure of oxygen–fraction of inspired oxygen less than or equal to 300 mm Hg; and critically ill cases were those that met any of respiratory failure criteria and required mechanical ventilation, or those with shock or other organ failure that required intensive care unit care.

### Study Design

This cohort study was conducted during the COVID-19 outbreak. A trained team of medical staff reviewed and collected the demographic, epidemiological, clinical, and laboratory data from electronic medical records. We classified the patients into 3 groups according to 3 levels of plasma K^+^—severe hypokalemia (<3 mmol/L), hypokalemia (3-3.5 mmol/L), and normokalemia (>3.5 mmol/L). We compared the clinical features, therapies, and treatment outcomes among the 3 groups, aiming to specify the association of hypokalemia with clinical features and to determine the underlying causes and clinical implications of hypokalemia. To elucidate the cause of hypokalemia, we established an experimental group of 20 patients with hypokalemia and a control group of 20 patients with normokalemia and measured their K^+^ in point urine samples. The association of gastrointestinal symptoms with plasma K^+^ concentrations was investigated. We randomly selected 3 patients with mild COVID-19 and 3 patients with severe COVID-19 with hypokalemia to investigate the treatment response to K^+^ supplements.

### Data Collection

The epidemiological investigation was focused on the transmission mode through the history of travel to or residence in Wuhan and the close contact with confirmed patients within 14 days.^9^ The etiological examinations included reverse transcriptase–polymerase chain reactions that detected SARS-CoV-2, influenza virus, respiratory syncytial virus, adenovirus, and parainfluenza virus in respiratory specimens. The immunological responses were evaluated by measuring white blood cell counts, lymphocyte counts, erythrocyte sedimentation rate, and C-reactive protein (CRP). Lungs, liver, renal, and cardiovascular functions were evaluated by laboratory tests, including coagulation profile, creatinine, blood urea nitrogen, alanine aminotransferase, aspartate transferase, creatine kinase (CK), lactate dehydrogenase (LDH), electrolytes, and arterial blood gas examination. Troponin was measured if patients exhibited myocardial damage, as indicated by elevated CK and CK–MB fraction. The level of K^+^ in urine samples was measured and reported in millimoles per gram of creatinine. Abnormal electrocardiogram (ECG) presentations involving the presence of U wave, ST-segment depression, and ventricular extrasystoles were recorded.

In addition, computed tomography was used to diagnose infections in the lungs. We retrieved therapeutic data and treatment outcomes from the electronic health records. Treatment outcomes were referred to as improved, cured, and failed.

### Statistical Analysis

We presented continuous variables as mean (SD). We performed the Kruskal-Wallis test for continuous variables. If the *P* value of the Kruskal-Wallis test was less than .05, we compared the means between each group using *t* tests. We presented categorical variables as number and percentage and compared proportions for categorical variables between groups using Fisher exact test. We considered 2-sided α < .05 to be statistically significant. All analyses were conducted with SPSS statistical software version 20.0 (IBM Corp). Data analysis was conducted in March 2020.

## Results

### Patients and Hypokalemia

During the study, 179 patients with COVID-19 were admitted to the hospital. One patient with severe renal failure, 2 patients with type 1 diabetes, and 1 patient with cancer were excluded from the study. The included 175 patients (mean [SD] age, 45 [14] years; age range, 15-85 years; 87 female patients [50%]) (Table 1) were classified has having severe hypokalemia (31 patients [18%]), hypokalemia (64 patients [37%]), and normokalemia (80 patients [46%]; 10 patients had plasma K^+^ >4 mmol/L) according to K^+^ concentrations at admission. No statistically significant difference was identified in terms of demographic features between the 3 groups. Seventy-one patients (41%) had underlying diseases, including hypertension (28 patients [16%]), diabetes (12 patients [7%]), and other conditions (31 patients [18%]). The prevalence of underlying diseases was associated with the severity of hypokalemia; 25 patients (81%) with severe hypokalemia and 29 patients (45%) with hypokalemia had underlying disease, whereas only 17 patients (12%) with normokalemia did (difference between hypokalemia and normokalemia, 33%; 95% CI, 20%-41%; *P* < .001).

**Table 1.** Demographic Characteristics and Clinical Symptoms of Patients, by Plasma Potassium Level

Characteristic	Patients, No. (%)				*P* value^a^			
	Total	Severe hypokalemia^b^	Hypokalemia^b^	Normokalemia^b^	*P*1	*P*2	*P*3	Kruskal-Wallis
No. of patients	175	31 (18)	64 (37)	80 (46)	NA	NA	NA	NA
Age, mean (SD), y	45 (14)	54 (13)	45 (14)	42 (14)	.003	<.001	.20	.001
Female	87 (50)	16 (52)	35 (55)	36 (45)	.80	.70	.30	NA
Temperature at admission, mean (SD), °C	37.2 (0.8)	37.6 (0.9)	37.2 (0.7)	37.1 (0.8)	.02	.005	.43	.002
Fever (>37 °C)	71 (41)	17 (55)	30 (47)	24 (30)	.50	.03	.06	NA
Dry cough	109 (62)	25 (81)	39 (61)	45 (56)	.06	.02	.60	NA
Dyspnea	23 (13)	8 (26)	9 (14)	6 (8)	.30	.02	.30	NA
Runny nose	8 (5)	1 (3)	3 (5)	4 (5)	>.99	>.99	>.99	NA
Sore throat	13 (7)	2 (6)	7 (11)	4 (5)	.70	.70	.20	NA
Diarrhea	35 (20)	9 (29)	16 (25)	10 (12)	.80	.05	.08	NA
Vomiting	7 (4)	1 (3)	5 (8)	1 (1)	.70	.50	.09	NA
Abdominal pain	5 (3)	1 (3)	2 (3)	2 (2)	>.99	>.99	>.99	NA
Myalgia	41 (23)	14 (45)	12 (19)	15 (19)	.01	.001	>.99	NA
Underlying disease	71 (41)	25 (81)	29 (45)	17 (21)	.002	.002	.001	NA

Abbreviation: NA, not applicable.

^a^ *P*1, *P*2, and *P*3 are the *t* test *P* values for comparisons between the severe hypokalemia and hypokalemia group, the severe hypokalemia and normokalemia group, and the hypokalemia and normokalemia group, respectively. Kruskal-Wallis is the *P* value for comparisons among all 3 groups.

^b^ Severe hypokalemia is defined as plasma potassium level less than 3 mmol/L, hypokalemia is defined as plasma potassium level 3 to 3.5 mmol/L, and normokalemia is defined as plasma potassium level greater than 3.5 mmol/L.

### Symptoms

Among 175 patients, 3 common symptoms were dry cough (109 patients [62%]), fever (71 patients [41%]), and diarrhea (35 patients [20%]), with 29% of patients with severe hypokalemia having diarrhea (Table 1). The diarrhea was generally mild, with a mean of 6 onsets per day, and lasted for 1 to 4 days. The prevalence of vomiting and abdominal pain ranged from 1% to 8%. All patients had a normal urinary volume of 2000 to 2500 mL per day. The patients with severe hypokalemia had statistically significantly higher body temperature (mean [SD], 37.6 °C [0.9 °C]) than the patients with hypokalemia (mean [SD], 37.2 °C [0.7 °C]; difference, 0.4 °C; 95% CI, 0.2-0.6 °C; *P* = .02) and the patients with normokalemia (mean [SD], 37.1 °C [0.8 °C]; difference, 0.5 °C; 95% CI, 0.3-0.7 °C; *P* = .005).

### Laboratory, Computed Tomography, and ECG Examinations

**Figure 1.**
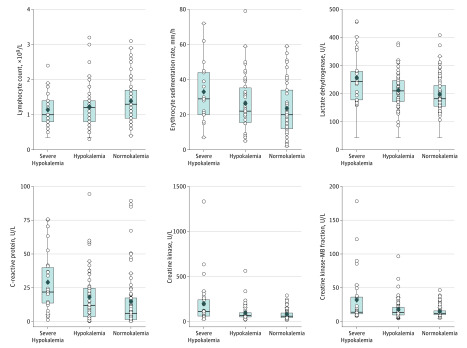
Distribution of Commonly Abnormal Indices Between Patients With Severe Hypokalemia, Hypokalemia, and Normokalemia Lines within boxes denote medians, diamonds denote means, tops and bottoms of boxes denote 75th and 25th percentiles, respectively, circles denote data for individual patients, and error bars denote 95% CIs. SI conversion factors: to convert creatine kinase to microkatals per liter, multiply by 0.0167; lactate dehydrogenase to microkatals per liter, multiply by 0.0167.

Among a series of laboratory examinations, the common abnormal indices included elevated CRP level (94 patients [54%]; mean [SD] 19 [21] mg/L; median, 12 mg/L; interquartile range [IQR], 3-24 mg/L), erythrocyte sedimentation rate (76 patients [43%]; mean [SD], 26 [16] mm/h), LDH level (52 patients [30%]; mean [SD], 214 [69] U/L; to convert LDH to microkatals per liter, multiply by 0.0167), CK–MB fraction (43 patients [25%]; mean [SD], 19 [20] U/L; median, 13 U/L; IQR, 10-16 U/L), and CK level (30 patients [17%]; mean [SD], 108 [131] U/L; median, 69 U/L; IQR, 50-112 U/L; to convert to microkatals per liter, multiply by 0.0167), as well as decreased lymphocyte count (71 patients [41%]; mean [SD], 1.28 [0.6] × 10^9^/L) and white blood cell count (53 patients [30%]; mean [SD], 4.9 [1.5] × 10^9^/L) (Figure 1 and Table 2). Patients with higher levels of hypokalemia also had higher CK levels (severe hypokalemia, mean [SD], 200 [257] U/L [median, 113 U/L; IQR, 61-242 U/L]; hypokalemia, mean [SD], 97 [85] U/L; and normokalemia, mean [SD], 82 [57] U/L), higher CK–MB fraction (severe hypokalemia, mean [SD], 32 [39] U/L [median, 14 U/L; IQR, 11-36 U/L]; hypokalemia, mean [SD], 18 [15] U/L; and normokalemia, mean [SD], 15 [8] U/L), higher LDH levels (mean [SD], severe hypokalemia, 256 [88] U/L; hypokalemia, 212 [59] U/L; and normokalemia, 199 [61] U/L), and higher CRP levels (severe hypokalemia, mean [SD], 29 [23] U/L; hypokalemia, 18 [20] U/L [median, 12 U/L; IQR, 4-25 U/L]; and normokalemia, mean [SD], 15 [18] U/L [median, 6 U/L; IQR, 3-17 U/L]).

**Table 2.** Laboratory Examinations and Treatments of Patients by Plasma Potassium Level

Characteristic (reference values)	Mean (SD)				*P* value^a^			
	Total	Severe hypokalemia^b^	Hypokalemia^b^	Normokalemia^b^	*P*1	*P*2	*P*3	Kruskal-Wallis
Patients, No. (%)	175	31 (18)	64 (37)	80 (46)	NA	NA	NA	NA
Laboratory examinations								
White blood cell count, × 10^9^/L (4-10 × 10^9^/L)	4.9 (1.5)	4.8 (1.4)	4.6 (1.6)	5.2 (2.1)	NA	NA	NA	.3
Decreased, patients, No. (%)	53 (30)	10 (32)	22 (34)	21 (26)	>.99	.60	.40	NA
Lymphocyte count, × 10^9^/L (1.1-3.2 × 10^9^/L)	1.28 (0.6)	1.14 (0.5)	1.24 (0.6)	1.39 (0.6)	NA	NA	NA	.1
Decreased, patients, No. (%)	71 (41)	19 (61)	25 (39)	27 (34)	.05	.01	.60	NA
C-reactive protein, mg/L (0-8 mg/L)	19 (21)	29 (23)	18 (20)	15 (18)	.02	.001	.34	<.001
Median (IQR)	12 (3-24)		12 (4-25)	6 (2-17)				
Increased, patients, No. (%)	94 (54)	24 (77)	37 (58)	33 (41)	.07	<.001	.06	NA
Erythrocyte sedimentation rate, mm/h (0-20 mm/h)	26 (16)	33 (16)	26 (16)	24 (15)	.05	.006	.44	.04
Increased, patients, No. (%)	76 (43)	18 (53)	29 (45)	29 (36)	.30	.05	.30	NA
Creatine kinase, U/L (55-170 U/L)^b^	108 (131)	200 (257)	97 (85)	82 (57)	.005	<.001	.21	.001
Median (IQR)	69 (50-112)	113 (61-242)						
Increased, patients, No. (%)	30 (17)	11 (35)	10 (16)	9 (11)	.04	.005	.50	NA
Creatine kinase–MB fraction, U/L (0-18 U/L)	19 (20)	32 (39)	18 (15)	15 (8)	.01	<.001	.12	.04
Median (IQR)	13 (10-16)	14 (11-36)						
Increased, patients, No. (%)	43 (25)	16 (52)	19 (30)	8 (10)	.04	<.001	.005	NA
Lactate dehydrogenase, U/L (40-240 U/L)	214 (69)	256 (88)	212 (59)	199 (61)	.005	<.001	.20	.002
Increased, patients, No. (%)	52 (30)	16 (52)	17 (27)	19 (24)	.02	.002	.40	NA
Alanine aminotransferase, U/L (<40 U/L)	32 (31)	44 (38)	29 (18)	28 (30)	.01	.02	.81	.02
Increased, patients, No. (%)	23 (13)	12 (39)	11 (17)	NA	.04	<.001	<.001	NA
Aspartate aminotransferase, U/L (<40 U/L)	30 (18)	37 (22)	29 (18)	28 (16)	.06	.02	.72	.03
Increased, patients, No. (%)	19 (11)	9 (29)	10 (16)	NA	.20	<.001	<.001	NA
Creatinine, μmol/L (25-110 μmol/L)	68 (31)	71 (17)	69 (31)	65 (33)	NA	NA	NA	.15
Blood urea nitrogen, mmol/L (3-7 mmol/L)^c^	3.8 (1.3)	3.9 (1.4)	3.7 (1.1)	3.6 (1.4)	NA	NA	NA	.70
pH (7.35-7.45)	7.41 (0.04)	7.42 (0.04)	7.40 (0.04)	7.40 (0.04)	NA	NA	NA	.40
pH >7.45, patients, No. (%)	19 (11)	9 (29)	5 (8)	5 (6)	.01	.003	.80	NA
CO_2_ pressure, kPa (4.4-6.3 kPa)	5.4 (0.8)	5.5 (0.7)	5.4 (0.9)	5.3 (0.8)	NA	NA	NA	.70
Decreased, patients, No. (%)	9 (5)	4 (13)	4 (6)	1 (1)	.40	.02	.20	NA
O_2_ saturation, % (93%-100%)	96.5 (2.3)	96.3 (2.6)	96.6 (2.0)	96.8 (2.1)	NA	NA	NA	.60
Decreased, patients, No. (%)	9 (5)	4 (13)	4 (6)	1 (1)	.40	.02	.20	NA
Potassium, mmol/L (3.5-5.5 mmol/L)	3.4 (0.4)	2.9 (0.1)	3.3 (0.1)	3.8 (0.3)	<.001	<.001	<.001	<.001
Sodium, mmol/L (135-145 mmol/L)	138 (3)	137 (3)	138 (3)	138 (3)	NA	NA	NA	.06
Decreased, patients, No. (%)	23 (13)	8 (26)	11 (17)	4 (5)	.40	.004	.03	NA
Chloride, mmol/L (96-105 mmol/L)	102 (3)	100 (4)	102 (3)	103 (3)	.008	<.001	.05	.01
Decreased, patients, No. (%)	5 (3)	2 (6)	2 (3)	1 (1)	.60	.20	.60	NA
Computed tomography and electrocardiogram examinations, patients, No. (%)								
Pulmonary infection	169 (97)	31 (100)	63 (98)	75 (94)	>.99	.30	>.99	NA
Abnormal electrocardiogram presentation	35 (20)	15 (48)	10 (16)	10 (12)	.001	<.001	.60	NA
Treatments and outcomes, patients, No. (%)								
Oxygen inhalation	51 (29)	20 (65)	20 (31)	11 (14)	.004	<.001	.04	NA
Interferon-α	170 (97)	31 (100)	62 (97)	77 (96)	>.99	>.99	>.99	NA
Lopinavir-ritonavir	151 (86)	30 (97)	57 (89)	64 (80)	.30	.40	.20	NA
Umifenovir	140 (80)	28 (90)	52 (81)	60 (79)	.40	.10	.40	NA
Glucocorticoid	12 (7)	7 (23)	5 (8)	NA	.05	<.001	.02	NA
Complication^d^	11 (6)	6 (19)	3 (5)	2 (2)	.05	.006	.70	NA
Severe cases	37 (21)	13 (42)	18 (28)	6 (8)	.20	<.001	<.001	NA
Critical cases	3 (2)	3 (10)	NA	NA	.03	.03	NA	NA

Abbreviation: NA, not applicable.

SI conversion factors: to convert alanine aminotransferase to microkatals per liter, multiply by 0.0167; aspartate aminotransferase to microkatals per liter, multiply by 0.0167; creatine kinase to microkatals per liter, multiply by 0.0167; lactate dehydrogenase to microkatals per liter, multiply by 0.0167.

^a^ *P*1, *P*2, and *P*3 are the *t* test *P* values for comparisons between the severe hypokalemia and hypokalemia group, the severe hypokalemia and normokalemia group, and the hypokalemia and normokalemia group, respectively. Kruskal-Wallis is the *P* value for comparisons among all 3 groups.

^b^ Severe hypokalemia is a plasma potassium level less than 3 mmol/L, hypokalemia is a plasma potassium level 3 to 3.5 mmol/L, and normokalemia is a plasma potassium level greater than 3.5 mmol/L.

^c^ All patients had normal blood urea nitrogen and creatinine concentrations.

^d^ Includes respiratory failure, sepsis, liver damage, respiratory distress, and cardiac damage.

Of 40 severely and critically ill patients, 34 (85%) had hypokalemia (Table 1, Table 2, and Figure 1). For 21 patients who had very elevated CK level and CK–MB fraction, their troponin was measured. One patient had elevated troponin (0.17 μg/L; reference range, 0-0.1 μg/L). Among 95 patients (54%) with K^+ ^less than 3.5 mmol/L, the mean (SD) plasma K^+^ in 31 patients exhibiting gastrointestinal symptoms was 3.12 (0.21) mmol/L, which was not meaningfully different from the mean (SD) value of 3.21 (0.21) mmol/L in 64 patients without gastrointestinal symptoms. Regarding the urinary K^+^ output, 20 patients with hypokalemia had a mean (SD) K^+^ concentration of 32 (11) mmol/g of creatinine, which was higher than the corresponding mean (SD) of 18 (7) mmol/g of creatinine for 20 patients with normokalemia (difference, 14 mmol/g; 95% CI, 10-18 mmol/g; *P* < .001). Concerning computed tomography images and ECG, 169 patients (97%) had a pulmonary infection, and 35 patients (20%) had abnormal ECG results, with the prevalences of abnormal ECG results of 48% for patients with severe hypokalemia, 16% for those with hypokalemia, and 12% for those with normokalemia.

### Treatment Outcomes

The common treatments involved interferon-α (170 patients [97%]), lopinavir-ritonavir (151 patients [86%]), umifenovir (140 patients [80%]), and oxygen inhalation (51 patients [29%]) (Table 2). Twelve patients (7%) were given glucocorticoid. No patients were given diuretics, bronchodilators, insulin, and other drugs that might cause hypokalemia. Of 40 severely or critically ill patients, 40% had severe hypokalemia (vs 11% of mildly and moderately ill patients; difference 29%; 95% CI, 21%-37%; *P* < .001) and 85% had hypokalemia (vs 45% of mildly and moderately ill patients; difference, 40%; 95% CI, 32%-49%; *P* < .001), both of which were statistically significant differences (Table 1 and Table 3). Compared with patients with mild-to-moderate disease, severely and critically ill patients also had a higher prevalence of abnormal ECG presentations (20 patients [14%] vs 15 patients [38%]), decreased white blood cell count (36 patients [27%] vs 17 patients [43%]), decreased lymphocyte counts (43 patients [32%] vs 28 patients [70%]), as well as elevated CK level (18 patients [13%] vs 12 patients [30%]), CK–MB fraction (23 patients [17%] vs 20 patients [50%]), LDH level (31 patients [23%] vs 21 patients [52%]), and CRP level (53 patients [39%] vs 28 patients [70%]). Among these abnormal indices, hypokalemia, elevated CRP, and decreased lymphocytes were the 3 most prevalent indices among severe and critically ill patients. Elevated alanine aminotransferase and aspartate aminotransferase were generally mild and came to normal levels after liver support therapy (Table 2 and Table 3).

**Table 3.** Incidence of Abnormal Features Between the Severely and Critically Ill Patients and Moderately and Mildly Ill Patients

Feature	Patients, No. (%)		*P* value
	Severely and critically ill (n = 40)	Moderately and mildly ill (n = 135)	
Potassium, mean (SD), mmol/L	3.2 (0.3)	3.5 (0.4)	.001
Potassium <3 mmol/L	16 (40)	15 (11)	<.001
Potassium 3-3.5 mmol/L	18 (45)	46 (34)	.30
Creatine kinase >170 U/L	12 (30)	18 (13)	.03
Creatine kinase–MB fraction >18 U/L	20 (50)	23 (17)	<.001
Lactate dehydrogenase >240 U/L	21 (52)	31 (23)	<.001
Oxygen saturation <93%	9 (22)	NA	NA
C-reactive protein >8 mg/L	28 (70)	53 (39)	.001
Alanine aminotransferase >40 U/L	10 (25)	13 (10)	.02
Aspartate transferase >40 U/L	10 (25)	9 (7)	.003
Leukopenia (<4 × 10^9^/L)	17 (43)	36 (27)	.08
Decreased lymphocyte count (<1.1 × 10^9^/L)	28 (70)	43 (32)	<.001
Abnormal electrocardiogram presentation	15 (38)	20 (14)	<.001

Abbreviation: NA, not applicable.

SI conversion factors: to convert alanine aminotransferase to microkatals per liter, multiply by 0.0167; aspartate aminotransferase to microkatals per liter, multiply by 0.0167; creatine kinase to microkatals per liter, multiply by 0.0167; lactate dehydrogenase to microkatals per liter, multiply by 0.0167.

**Figure 2.**
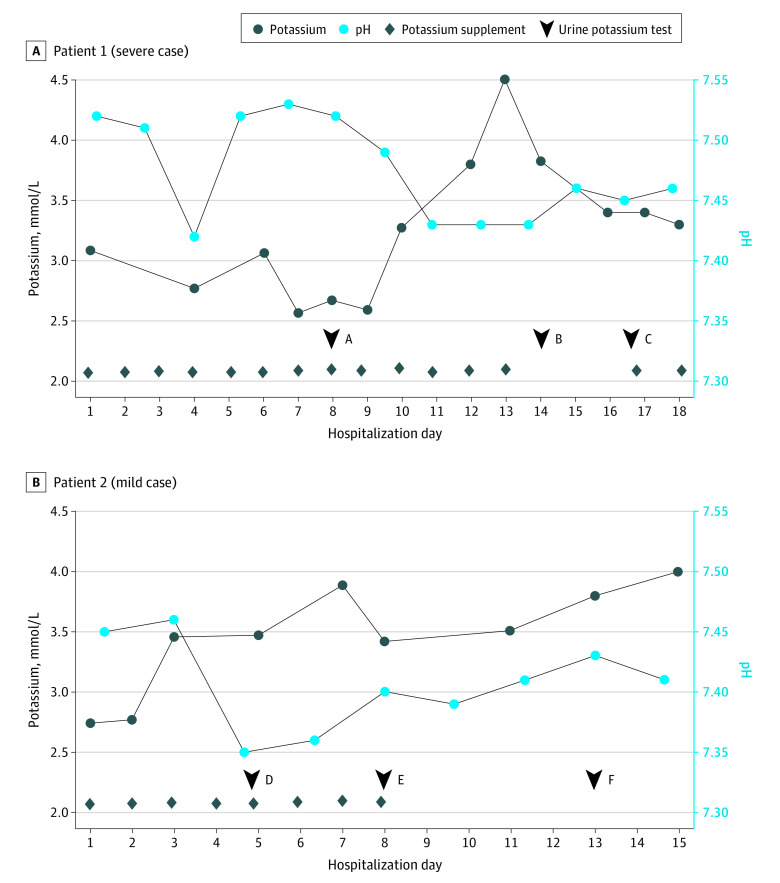
Trend in Plasma Potassium and pH, and the Response to Potassium Supplement for 2 Patients With Coronavirus Disease 2019 (COVID-19) A, Patient with a severe case of COVID-19. The failure of potassium supplements is associated with the increased loss of urinary potassium. The urinary potassium levels were 41, 50, and 38 mmol/g of creatinine at points A, B, and C, respectively. B, Patient with a mild case of COVID-19. The loss of urinary potassium is relieved, ensuring the effective treatment by potassium supplements. Urinary potassium levels were 32, 21, and 9 mmol/g of creatinine at time points D, E, and F, respectively.

With regard to the therapy and treatment outcomes (Table 2), among 40 severely and critically ill patients, 16 (40%) had severe hypokalemia, 18 (28%) had hypokalemia, and 6 (8%) had normokalemia. The likelihood of being administered oxygen increased along with the severity of hypokalemia: 20 patients (65%) with severe hypokalemia, 20 patients (31%) with hypokalemia, and 11 patients (14%) with normokalemia were administered oxygen. Nearly all patients (170 patients [97%]) were given interferon-α, whereas the use of additional antiviral drugs was more common in the hypokalemia group, including 87 patients (92%) who received lopinavir-ritonavir and 80 patients (85%) who received umifenovir. With regard to the K^+^ supplements, the dosage for the patients with severe hypokalemia was 3 g (40 mEq) potassium chloride per day, for a total mean (SD) of 34 (4) g (mean [SD] 453 [53] mEq) potassium chloride during the whole hospital stay for each patient. Three mildly ill patients with hypokalemia achieved normokalemia after 5 to 8 days of receiving K^+^ supplements, whereas 3 severely ill patients with hypokalemia needed K^+^ supplements for 10 to 14 days to have steady normokalemia (Figure 2). With regard to pH, values greater than 7.45 were seen in 29% of patients with severe hypokalemia.

## Discussion

This study describes the high prevalence of hypokalemia among patients with COVID-19 and the positive association of the degree of hypokalemia with the severity of some symptoms of COVID-19. These findings suggest that hypokalemia was more attributable to renal loss of K^+^ than gastrointestinal loss.

Previous literature^10,11^ has shown that sufficient and appropriate plasma levels of K^+^ play a protective role in preventing myocardial failure through weakening cellular hyperpolarity and depolarization. SARS-CoV-2 causes heart dysfunction via the intensively expressed ACE2 in the patients’ myocardial cells that act as receptors for this virus. It is beneficial to patients that plasma K^+^ levels be frequently checked and maintained between 4.0 and 5.5 mmol/L.^12^ In the present study, hypokalemia was prevalent among the patients with COVID-19, because 54% (95 of 175) of patients had plasma K^+^ less than 3.5 mmol/L. Only 10 patients had plasma K^+^ greater than 4 mmol/L, which is the recommended concentration for patients with myocardial dysfunction. The low prevalence of optimal concentrations of plasma K^+^ implied a lack of a protective role of K^+^ for the patients’ hearts. Because COVID-19 is an emerging infectious disease, few data are available on serum K^+^ levels in patients with COVID-19. A previous study^13^ of 41 patients reported 4.2 mmol/L as the mean serum K^+ ^level and found higher K^+^ levels in patients in the intensive care unit (mean, 4.6 mmol/L) than in patients not in the intensive care unit (mean, 4.1 mmol/L), suggesting that the elevated serum K^+^ was associated with severity of illness. However, our findings, which were derived from more patients, contradict those of the previous study. Considering the implications of serum K^+^ concentrations in this disease, further investigation is necessary.

The present study also found that the degree of hypokalemia was associated with some clinical features that reflected the severity of the disease, including underlying conditions, high body temperature, and, notably, the elevated laboratory indices reflecting myocardial injuries, such as CK, CK–MB fraction, LDH, and abnormal ECG results. Regarding the blood gas results, the higher prevalence (29%) of pH of over 7.45 was seen in patients with severe hypokalemia because severe hypokalemia led to alkalosis due to hydrogen-potassium exchange between intracellular and extracellular fluid.^14^ Nevertheless, the prevalence of abnormal oxygen saturation and CO_2_ pressure were not sensitive enough to identify the difference between patients with different K^+^ levels. The patients who showed renal dysfunction were rare, according to a generally normal concentration of blood urea nitrogen and creatinine, as well as sufficient urine output. Because of the variations among laboratories and assays that may generate different reference values, it is not recommended to compare results directly. The current study provides the number and percentage of several abnormal laboratory results, which were considered to be essential indices for hypokalemia, myocardial injury, and evaluation of disease severity. We expect that these arrangements of results might facilitate comparison.

Because hypokalemia was prevalent among patients with COVID-19 and was associated with the severity of the disease, elucidation of the mechanisms for hypokalemia was necessary for the treatment of COVID-19. In the current situation, 2 possible causes of hypokalemia are increased gastrointestinal and urinary loss.^15^ Both causes can be possible in patients with COVID-19. However, the present findings indicate that gastrointestinal loss might not contribute much to hypokalemia for the following reasons: only a small proportion of patients with hypokalemia had gastrointestinal symptoms (eg, 29% of patients with severe hypokalemia had diarrhea); among the patients with hypokalemia, there were no differences between those with and those without diarrhea; and most patients had mild diarrhea with a mean number of 6 onsets per day and a short duration. Therefore, hypokalemia might primarily result from increased urine loss. This was shown in this study by the increased urinary K^+^ output in the hypokalemia group compared with the control group with normokalemia. This finding that increased urinary K^+^ was the primary cause of hypokalemia was consistent with the pathogenesis of SARS-CoV-2. In a healthy person, RAS activity is balanced by ACEI (which increases RAS activity) and ACE2 (which decreases RAS activity). When SARS-CoV-2 binds and degrades ACE2, the ability of ACE2 to regulate RAS is reduced and it cannot antagonize ACEI.^4^ The final result is RAS activity is increased, which acts like secondary increased aldosterone.^16^ Increased RAS activity enhances the distal delivery of sodium and water to collecting tubule of the kidney and the excretion of potassium, because previous literature also has shown that serum K^+^ is negatively associated with plasma renin activity.^7^

Because hypokalemia can affect myocardial function, timely treatment is necessary to achieve good outcomes. However, the mechanism of hypokalemia in the present study suggested that it was challenging to achieve normokalemia in the presence of continuous renal loss of K^+^. The ongoing K^+^ supplements often failed to correct hypokalemia when the urinary loss of K^+^ persisted in the patients with severe disease. The steady normalization of serum potassium by use of potassium supplements at the later phase of hospitalization occurred at the same time as improvement of disease. We interpreted that this phenomenon might be associated with less renal loss of potassium, which suggested the patients had a more balanced RAS function, as the counter-function of ACE2 was recovered to some degree.

Although COVID-19 causes injury to lungs, heart, liver, and kidney, our study also found that the occurrence of abnormal indices of heart, liver, and kidney was low (Table 2). Several laboratory indices, such as elevated CK, CK–MB fraction, LDH, alanine aminotransferase, and aspartate aminotransferase, usually came to normal levels or substantially improved after relevant treatment. The superficial mildness contradicted with the sudden progression of disease in some patients. This contradiction might result from the fact that the biomarkers were not sensitive enough to reflect the underlying progression of this disease. A more sensitive biomarker can facilitate monitoring of ongoing COVID-19. As discussed already, 85% of severely and critically ill patients had hypokalemia (Table 3), showing that depletion of K^+^ was prevalent. On the basis of the analysis of the trend in plasma K^+^ and urine output of K^+^, the end of the depletion often suggested a good prognosis (Figure 2). Hence, a comprehensive analysis of K^+^ depletion can be achieved by monitoring urinary K^+^ loss, plasma K^+^, and the response to K^+^ supplement treatment. Importantly, the current knowledge about hypokalemia directly reflects the very basis of the pathogenesis of SARS-CoV-2 and might be a reliable, timely, and sensitive biomarker to reflect the progression of COVID-19.

### Limitations

This study has some limitations. There are several risk factors associated with the severity of COVID-19. The confounding factors may affect the results. However, as the present findings reflect the fundamental pathophysiological mechanism of COVID-19 and the great proportion of patients with hypokalemia, we believe that our conclusion is valid.

## Conclusions

The present study has identified the prevalence of hypokalemia in patients with COVID-19. The correction of hypokalemia is challenging because of continuous renal loss of K^+ ^resulting from the degradation of ACE2 by the binding of SAR-CoV-2. The end of the loss of K^+^ may be a reliable, timely, and sensitive biomarker that reflects the end of disruption on the RAS system by SAR-CoV-2. Because of the possible effects on cardiovascular functions, neurohormonal activation, and other vital organs by hypokalemia, clinicians should pay attention to hypokalemia and the patients’ response to K^+^ supplements.
